# HIV- and sex work-related stigmas and quality of life of female sex workers living with HIV in South Africa: a cross-sectional study

**DOI:** 10.1186/s12879-022-07892-4

**Published:** 2022-12-06

**Authors:** Claire Chen, Stefan Baral, Carly A. Comins, Mfezi Mcingana, Linwei Wang, Deliwe Rene Phetlhu, Ntambue Mulumba, Vijay Guddera, Katherine Young, Sharmistha Mishra, Harry Hausler, Sheree R. Schwartz

**Affiliations:** 1 Department of Epidemiology, Johns Hopkins Bloomberg School of Public Health, 615 N. Wolfe St, W3503, Baltimore, MD 21205 USA; 2 TB HIV Care Association, Cape Town, South Africa; 3 MAP-Centre for Urban Health Solutions, St. Michael’s Hospital, Unity Health Toronto, Toronto, ON Canada; 4 University of Western Cape, Cape Town, South Africa; 5 Department of Medicine, Division of Infectious Disease, University of Toronto, Toronto, ON Canada; 6 Institute of Health Policy, Management, and Evaluation, University of Toronto, Toronto, ON Canada

**Keywords:** Female sex workers, Stigma, Quality of life, South Africa, HIV, Environmental domain

## Abstract

**Background:**

Environmental quality of life (QoL) assesses individually perceived factors such as physical safety and security, accessibility, quality of healthcare, and physical environment. These factors are particularly relevant in the context of sex work and HIV, where stigma has been identified as an important barrier across several prevention and treatment domains. This study aims to examine the association between different types of HIV- and sex work-related stigmas and environmental QoL among female sex workers (FSW) living with HIV in Durban, South Africa.

**Methods:**

We conducted cross-sectional analyses using baseline data from the *Siyaphambili* randomized controlled trial. FSW who reported sex work as their primary source of income and had been diagnosed with HIV for ≥ 6 months were enrolled from June 2018–March 2020, in eThekwini, South Africa. We evaluated the association between environmental QoL, dichotomizing the environmental domain score collected by the WHO Quality of Life HIV Brief (WHOQOL-HIV BREF) questionnaire at the median, and stigma using modified robust Poisson regression models. Five stigma subscales were assessed: sex work-related (anticipated, enacted, or internalized stigma) and HIV-related (anticipated or enacted stigma).

**Results:**

Among 1373 FSW, the median environmental QoL was 10.5 out of 20 [IQR: 9.0–12.5; range 4.0–19.0], while the median overall QoL was 3 out of 5 [IQR: 2–4; range 1–5]. One-third of FSW (n = 456) fell above the median environmental QoL score, while 67% were above the median overall QoL (n = 917). Reporting anticipated sex work stigma was associated with lower environmental QoL (adjusted prevalence ratio [aPR] 0.74 [95% CI 0.61, 0.90]), as was severe internalized sex work stigma (aPR: 0.64, 95% CI 0.48, 0.86). Reporting enacted HIV stigma versus none was similarly associated with lower environmental QoL (aPR: 0.65, 95% CI 0.49, 0.87). Enacted sex work stigma and anticipated HIV stigma were not statistically associated with environmental QoL.

**Conclusions:**

This study highlights the need to consider the impact of multiple stigmas on FSW’s non-HIV related clinical outcomes, including safety and physical well-being. Moreover, these results suggest that addressing underlying structural risks may support the impact of more proximal HIV prevention and treatment interventions.

*Trial registration* NCT03500172 (April 17, 2018)

**Supplementary Information:**

The online version contains supplementary material available at 10.1186/s12879-022-07892-4.

## Background

Sex workers experience a high burden of human immunodeficiency virus (HIV) infection. Nearly one in ten sex workers globally are estimated to be living with HIV despite advances in HIV prevention and treatment, and HIV prevalence among sex workers is expected to rise, even as population-level HIV prevalence decreases [1]. In South Africa, an estimated 60% of female sex workers (FSW) are living with HIV, with less than half of FSW living with HIV estimated to be on antiretroviral therapy (ART) [2, 3]. Sex work- and HIV-related stigmas are sources of disparities in HIV prevalence and treatment outcomes [4].

Stigma takes on various forms, some of which cannot be directly observed or measured. Link and Phelan describe stigma as a social psychological phenomenon with four components: (1) distinguishing and labeling (or marking) differences, (2) assigning these differences negative attributes, (3) separating “us” from “them”, and (4) status loss and discrimination [5]. Stigma is highly culture- and power-dependent, leading to the societal marginalization of certain populations [6]. In the context of research and programs, stigma has often been grouped in categories including enacted, anticipated, and internalized stigmas. Enacted stigma refers to the actual experience of discrimination based on a stigmatized attribute [7]. Anticipated stigma is the expectation of stigmatization, whether or not the event actually occurs [8]. Internalized stigma is a form of stigma where one accepts negative attributes applied to themselves [9].

FSW living with HIV can experience stigmas related to both their positive HIV status and their occupation. The legal and political environments contributing to the criminalization of sex work and the dual stigmas associated with sex work and HIV serve as barriers to HIV treatment and prevention, linkage to ART, and retention in care among FSW [10]. Increased HIV-related stigma is also associated with lower ART adherence and higher viral load, as well as mental and physical health outcomes [11–14].

Quality of life (QoL) evaluates the perception of aspects of life ranging from individual health to community-level conditions [15]. Environmental QoL, as assessed by the World Health Organization (WHO), considers factors such as physical safety and security, financial resources, and access to healthcare [16]. It is well-documented that HIV-related stigma and discrimination negatively affect overall QoL [17–21], and previous literature has also reported a negative correlation between HIV-related stigma and environmental QoL [22, 23]. However, few studies examine these relationships among FSW or explore sex work-related stigma.

Addressing HIV-related stigma is a common research motive to reduce stigmatization via tactics such as improving provider interactions with patients [24] or targeting stigma in the larger community, such as among policymakers or law enforcement authorities [20]. This fails to recognize that multiple stigmatized identities may affect health-related outcomes [24]. FSW living with HIV are marginalized due to their dual identities of being a sex worker and living with HIV [1]. To appropriately assess the overall impact of stigma on QoL, it is necessary to examine stigma’s multiple sources.

Stangl and Earnshaw’s Health Stigma and Discrimination Framework was utilized to contextualize the stigmatization process [25]. Within this framework, stigmas resulting from HIV and sex work “marking” manifest into different stigma experiences or practices, which subsequently affect outcomes such as HIV morbidity, mortality, and QoL. This paper builds upon existing research and identifies potential areas on which to focus in overcoming the impact of multiple stigmas on QoL. The objective of this study is to describe QoL and stigma among FSW in Durban, South Africa, and assess the association between various HIV- and sex work-related stigmas and environmental QoL.

## Methods

### Study design and setting

These analyses utilize the baseline data of the ongoing *Siyaphambili* randomized controlled trial in eThekwini (Durban), South Africa. The study design and methods were detailed previously [26]. In summary, the intent of *Siyaphambili* is to investigate the impact and cost-effectiveness of two ART interventions (decentralized ART provision and individualized case management) alone and in combination among FSW living with HIV. The study is embedded within an established HIV prevention and treatment program for FSW operated by TB HIV Care, a non-profit organization.

Cisgender FSW 18 years and older were reached at sex work venues and eligible for study participation if they were diagnosed with HIV at least six months prior to study enrollment, non-pregnant, and engaged in sex work as their main source of income. This analysis used cross-sectional data from the baseline questionnaire, administered in private by research study staff in either English or isiZulu at enrollment.

### Measures

The outcome of interest, QoL, was assessed using the WHOQOL-HIV BREF questionnaire, which was previously validated in South Africans with HIV [27]. The survey consists of 31 questions on a 5-point Likert scale evaluating the respondent’s perception of their QoL in the past two weeks, ranging from “Very poor” (1-point) to “Very good” (5-points). The questions were developed to align with the WHOQOL Group’s definition of QOL as “an individual’s perception of their position in life in the context of the culture and value systems in which they live, and in relation to their goals, expectations, standards and concerns” [28]. Accordingly, the survey examines individuals’ satisfaction with various aspects of their day-to-day lives, such as living conditions, interpersonal interactions, and their physical and mental capacities. It assesses QoL in the physical (four items), psychological (five items), level of independence (four items), social relationships (four items), environment (eight items), and spirituality/religion/personal beliefs (SRPB, four items) domains. The remaining two items evaluate the respondent’s overall QoL and general health perceptions. Domain scores ranging from 4 to 20 were calculated by taking the average score of all items in each domain and multiplying by 4 to standardize the score across domains [16]. A higher score indicates a more positive impression of QoL [16]. If participants were missing data, the average scores were prorated based on the total items per domain. The scores were then dichotomized using the median of the possible score range (lower QoL < 12, higher QoL ≥ 12) [29, 30]. The participants’ response for the single item assessing overall QoL (“How would you rate your quality of life?”) was also dichotomized (low QoL < 3, high QoL ≥ 3). The Pearson’s correlation coefficient was calculated to be 0.44 between this response and the sum of the six QoL domain scores, indicating a moderate positive correlation as a measure of overall QoL. The Cronbach’s alpha of the six domains ranged from 0.42 (Physical) to 0.72 (Social Relationships) (Additional file 2: Table S1).

The baseline questionnaire included questions related to enacted, anticipated, and internalized stigma (a full list of the items is available in Additional file 1: Appendix Table A). Enacted stigma consisted of 10 closed (Yes/No) items for sex work-related stigma and seven closed items for HIV-related stigma. There were nine closed items for anticipated stigma (four HIV and five sex work). The enacted and anticipated stigma items were initially developed through literature reviews and in collaboration with key stakeholders, and variations have been adapted in several other studies [31–34]. More recently, the reliability of the sex work-related items was demonstrated among FSW in Togo and Burkina Faso via principal factor analysis [34]. Internalized stigma was evaluated for sex work only, using three 5-point Likert scale items (responses ranging from “Strongly Agree” to “Strongly Disagree”).

For each stigma subscale for sex work and separately for HIV (sex work enacted stigma, HIV enacted stigma, sex work anticipated stigma, HIV anticipated stigma and sex work related internalized stigma), the responses were further categorized. Enacted and anticipated stigma were organized into either no stigma (respondent answered “No” to all items) or any stigma (respondent answered “Yes” to at least one item). The internalized stigma scores were coded with responses ranging from 1 to 5 for each question (Strongly Agree = 1 to Strongly Disagree = 5). After the summed internalized stigma scores were prorated for missing responses, they were categorized into levels of no to minimal stigma (3–6), mild (7–9), moderate (10–12), or severe (13–15) stigma based on the score distribution among the study population.

Potential confounding variables were determined based on previous literature [17, 35–37]. Age, participants’ monthly income (measured in the South African rand currency, ZAR), and the length of HIV diagnosis were categorized based on the distribution of the data. Other covariates were education level (no formal education, some primary or secondary education, secondary education completed or higher) and employment status (sex work only versus employment outside of sex work). There was limited heterogeneity in employment (97% employed exclusively in sex work) and race (97% of FSW identified as Black), thus these were not included in the final multivariable models.

### Statistical analyses

It was hypothesized a priori that the study participants would experience lower QoL in the environment domain compared to other domains, due to the criminalized nature of sex work and associated external stressors [38]. A preliminary one-way analysis of variance between the six standardized QoL domain scores confirmed a statistically significant difference in mean score between the environment domain and the other five domains (Additional file 2: Table S2). The environment domain’s Cronbach’s alpha is 0.70, which is on the higher end of the range for all domains and is generally deemed acceptable reliability [39]. Given these results in combination with environment being a major point of interest, we focused specifically on environmental QoL (Additional file 1: Appendix Table B). Overall QoL was assessed as a secondary outcome, along with the distribution of the five stigma subscale scores and the environmental and overall QoL scores.

A Poisson regression with a robust variance estimator was used to estimate prevalence ratios (PRs) for all univariable stigma relationships with environmental QoL [40]. We chose to estimate prevalence ratios instead of odds ratios due to the high prevalence of the outcome (33% of the study population had higher environmental QoL). Additionally, modified robust Poisson regression was chosen over log-binomial due to failure of convergence of the latter model [41]. Similarly, we used modified robust Poisson univariable models to estimate the crude PR of higher overall QoL against each of the categorical stigma subscales.

We developed a multivariable model estimating the relative prevalence of environmental QoL, incorporating the five stigma subscales and adjusting for potential confounding variables. An analogous model was also developed using the dichotomized overall QoL outcome. The variance inflation factor (VIF) was calculated for the multivariable models to evaluate collinearity between the stigma subscales. The VIF did not exceed 10 (max observed: 1.32) among the stigma subscales in either the environmental or overall QoL model. Thus, all five subscales were retained in the final multivariable models.

Collapsing the environmental and overall QoL scores into binary variables could result in a loss of information or distortion of the true underlying association [42]. As a sensitivity analysis, a multiple linear regression analysis was performed for both outcomes. Multivariable modified robust Poisson models were also applied to the remaining five QoL domains to assess whether similar trends were observed compared to the environment domain.

Multiple imputation by chained equations was used to address missing covariate data in the primary and secondary analyses [43]. All analyses were performed using R version 4.0.2. A p-value of 0.05 was used to guide interpretation of statistical significance.

## Results

### Descriptive statistics

From June 22, 2018 to March 23, 2020, 1654 participants were screened for the *Siyaphambili* study, and 1391 participants were subsequently enrolled. After excluding participants who did not have baseline questionnaire data (n = 14) or did not have any QoL outcome data (n = 4), the total sample size included in this analysis was 1373 women. The overall mean scores for the six QoL domains were: (1) Physical: 13.54 (SD: 3.44); (2) Psychological: 13.76 (SD: 2.69); (3) Level of Independence: 13.79 (SD: 2.87); (4) Social Relationships: 12.76 (SD: 3.40); (5) Environment: 10.54 (SD: 2.73); and (6) SRPB: 13.61 (SD: 3.41) (Additional file 2: Table S1).

Table 1 presents the population’s sociodemographic and health-related characteristics. The mean age of the study participants was 32 years (SD: 8). Approximately 14% of the participants were newly diagnosed with HIV (< 1 year), the mean CD4 count was 572/μl (SD: 342), and 38% of the study population was virally suppressed per the South Africa Department of Health standards (< 50 copies/ml) [44]. Ninety-eight percent of women were born in South Africa.

**Table 1:** Characteristics of female sex workers living with HIV in Durban, South Africa (n = 1373)

	Total (n = 1373)	Higher environmental QoL (n = 455)	Lower environmental QoL (n = 918)	p-value
Age				0.850
Mean (SD)	32 (± 8.0)	32 (± 7.6)	32 (± 8.1)	
Race				0.419
Black	1335 (97%)	442 (97%)	893 (97%)	
Colored^a^	35 (3%)	13 (3%)	22 (3%)	
Indian	3 (0%)	0 (0%)	3 (0%)	
Length of HIV Diagnosis (years)^b^				0.982
≤ 1	194 (14%)	67 (15%)	127 (15%)	
> 1 and ≤ 5	517 (39%)	174 (39%)	343 (38%)	
> 5 and ≤ 10	341 (26%)	112 (25%)	229 (26%)	
> 10	278 (21%)	94 (21%)	184 (21%)	
CD4 count (/μl)^c^				0.112
≤ 200	165 (13%)	49 (12%)	116 (14%)	
201–350	210 (17%)	62 (15%)	148 (17%)	
351–500	257 (21%)	100 (23%)	157 (19%)	
> 500	661 (49%)	218 (50%)	443 (50%)	
Viral Load (copies/mL)^d^				0.008
< 50	518 (38%)	198 (43%)	320 (36%)	
50–1000	208 (15%)	67 (15%)	141 (15%)	
> 1000	642 (47%)	190 (42%)	452 (49%)	
On ART^e^				0.091
No	180 (14%)	50 (12%)	130 (16%)	
Yes	1151 (86%)	397 (88%)	754 (84%)	
Education level^f^				0.013
No formal education	25 (2%)	8 (2%)	17 (2%)	
Some primary or secondary education	1099 (80%)	342 (76%)	757 (82%)	
Secondary education completed or higher	242 (18%)	99 (22%)	143 (16%)	
Employment status^g^				0.074
Sex work only	1288 (94%)	419 (92%)	869 (95%)	
Employment outside of sex work	43 (3%)	21 (5%)	22 (2%)	
Other	41 (3%)	15 (3%)	26 (3%)	
Relationship status				0.008
Single	696 (51%)	211 (46%)	485 (53%)	
Steady partner, living together	208 (15%)	63 (14%)	145 (16%)	
Steady partner, not living together	469 (34%)	181 (40%)	288 (31%)	
Monthly income (ZAR)^h^				< 0.001
< 1500	431 (32%)	112 (26%)	319 (36%)	
≥ 1500 and < 3000	438 (33%)	141 (32%)	297 (33%)	
≥ 3000	469 (35%)	188 (42%)	281 (31%)	

^a^Colored refers to an officially designated multiracial ethnic group native to South Africa

^b^Forty-three participants were missing a response for length of HIV diagnosis

^c^Eighty participants were missing a response for CD4 count

^d^Five participants were missing a response for viral load

^e^Forty-two participants were missing a response for on ART

^f^Seven participants were missing a response for education level

^g^One participant was missing a response for employment status

^h^Thirty-five participants were missing a response for monthly income

Table 2 presents the stigma subscale scores across both sex work and HIV attributes. Thirty-one percent of participants (n = 496) reported any anticipated sex work stigma, 67% (n = 913) experienced any enacted sex work stigma, and 59% (n = 814) reported internalized sex work stigma (score ≥ 7). For HIV-related stigmas, 115 (8%) participants reported any anticipated stigma, and 243 (18%) experienced any enacted stigma.

**Table 2:** Prevalence of stigma in female sex workers living with HIV in Durban, South Africa (n = 1373)

Attribute	Stigma subscale	Experienced any stigma
Sex Work	Anticipated	496 (36%)
	Enacted	913 (67%)
	Internalized	
	None/minimal (3–6)	558 (41%)
	Mild (7–9)	304 (22%)
	Moderate (10–12)	356 (26%)
	Severe (13–15)	155 (11%)
HIV	Anticipated	115 (8%)
	Enacted	243 (18%)

### Univariable analysis

Based on the univariable analysis, there was a statistically significant association between each of the five stigma subscales and the environment domain of QoL (Table 3). For the enacted and anticipated stigmas for both HIV and sex work, there was a statistically significant decrease in environmental QoL associated with each of the four stigma domains. The groups with moderate and severe internalized sex work stigma had statistically significantly lower prevalence of high environmental QoL compared to the reference group with no to minimal internalized stigma.

**Table 3:** Crude prevalence ratios of high quality of life in FSW living with HIV (n = 1373)

Attribute	Stigma subscale	Higher environment QoL		Higher overall QoL	
		Crude PR (95% CI)	p-value	Crude PR (95% CI)	p-value
Sex work	Any anticipated (ref: no anticipated)	0.65 (0.54, 0.77)	< 0.001^*^	0.92 (0.85, 0.99)	0.035^*^
	Any enacted (ref: no enacted)	0.76 (0.65, 0.88)	< 0.001^*^	0.89 (0.83, 0.96)	0.002^*^
	Internalized None/minimal (ref: 3–6)	Ref		Ref	
	Mild (7–9)	0.93 (0.76, 1.11)	0.418	1.08 (0.99, 1.18)	0.083
	Moderate (10- 12)	0.77 (0.63, 0.94)	0.009^*^	0.88 (0.80, 0.98)	0.016^*^
	Severe (13–15)	0.64 (0.47, 0.86)	0.003^*^	0.89 (0.78, 1.03)	0.108
HIV	Any Anticipated (ref: no anticipated)	0.58 (0.40, 0.85)	0.005^*^	0.76 (0.63, 0.91)	0.003^*^
	Any Enacted (ref: no enacted)	0.55 (0.42, 0.72)	< 0.001^*^	0.78 (0.69 0.89)	< 0.001^*^

Prevalence ratios were estimated using modified robust Poisson regression

^*^p-value < 0.05

The univariable analyses were also conducted with the dichotomized score for the overall QoL. Again, the groups reporting any stigma in the enacted and anticipated stigma subscales for HIV and sex work had a statistically significant decrease in overall QoL compared to the reference groups of no stigma. Among the grouped scores for internalized sex work stigma, the group with moderate stigma experienced a statistically significantly lower prevalence of higher overall QoL compared to the reference group.

### Multivariable analysis

After adjusting for sociodemographic and clinical covariates and including all five stigma subscales in the multivariable analysis, three stigmas were statistically significantly associated with environmental QoL (Table 4). Women who experienced any anticipated sex work stigma were 26% less likely to report high environmental QoL compared to those who did not report any anticipated stigma (aPR: 0.74, 95% CI 0.61, 0.90). FSW with moderate and severe internalized sex work stigma had lower environmental QoL compared to those with no to minimal stigma (moderate aPR: 0.75, 95% CI 0.62, 0.91; severe aPR: 0.64, 95% CI 0.48, 0.86). Women reporting any enacted HIV stigma had a 35% lower prevalence of high QoL 0.65 (95% CI 0.49, 0.87) versus women with no enacted HIV stigma experience.

**Table 4:** Adjusted prevalence ratios of higher quality of life in FSW living with HIV (n = 1373)

Attribute	Stigma subscale	Higher environment QoL		Higher overall QoL	
		aPR^*^ (95% CI)	p-value	aPR^*^ (95% CI)	p-value
Sex Work	Any anticipated (ref: no anticipated)	0.74 (0.61, 0.90)	0.002^**^	1.00 (0.92, 1.10)	0.918
	Any enacted (ref: no enacted)	0.90 (0.76, 1.06)	0.191	0.91 (0.84, 0.99)	0.041^**^
	Internalized None/minimal (ref: 3–6)	Ref		Ref	
	Mild (7–9)	0.91 (0.76, 1.10)	0.323	1.05 (0.96, 1.15)	0.251
	Moderate (10–12)	0.75 (0.62, 0.91)	0.003^**^	0.87 (0.78, 0.96)	0.006^**^
	Severe (13–15)	0.64 (0.48, 0.86)	0.003^**^	0.90 (0.78, 1.02)	0.108
HIV	Any anticipated (ref: no anticipated)	0.90 (0.60, 1.35)	0.621	0.86 (0.71, 1.04)	0.119
	Any enacted (ref: no enacted)	0.65 (0.49, 0.87)	0.004^**^	0.85 (0.75, 0.97)	0.016^**^

Adjusted prevalence ratios (aPR) were estimated using modified robust Poisson regression

^*^Adjusted for age, level of education, monthly income, length of HIV diagnosis

^**^p-value < 0.05

In the multivariable analysis for overall QoL, women who experienced any enacted sex work stigma had an aPR of 0.91 (95% CI 0.84, 0.99) compared to those who reported no enacted stigma. FSW with moderate internalized sex work stigma had an aPR of 0.87 (95% CI 0.78, 0.96) of higher overall QoL compared to those with no or minimal stigma. Any enacted HIV stigma was also statistically significantly associated with lower prevalence of higher overall QoL (aPR: 0.85, 95% CI 0.75, 0.97) versus the group with no enacted stigma.

The relationship between stigma domains and environmental and overall QoL were similar in sensitivity analyses using continuous rather than dichotomized QoL outcomes (Additional file 2: Table S3). Furthermore, the sensitivity analyses showed similar relationships between stigma and the remaining five QoL domains (physical, psychological, level of independence, social relationships, and SRPB) (Additional file 2: Table S4).

## Discussion

This analysis explores stigma dynamics among sex workers living with HIV and goes beyond behavioral, clinical, or interpersonal outcomes in existing research. Importantly, it identifies stigmas that are associated with sex workers’ environmental QoL, namely physical environment, perceived safety, and ability to meet every day needs. In this study, anticipated sex work stigma, internalized sex work stigma, and enacted HIV stigma are negatively associated with environmental QoL. These data show that in addition to HIV-related stigmas, sex work-related stigmas also factor into the QoL of FSW living with HIV. Together these findings highlight the need for HIV interventions to consider sex work stigma drivers and the environmental aspects of FSW’s wellbeing.

Although it is well documented that sex workers have increased vulnerability for HIV infection [45] and are marginalized in HIV prevention and treatment efforts [4], this study contributes new findings of how HIV-related stigmas affect the environmental and overall QoL of FSW. The negative associations correspond with existing literature linking HIV-related stigma with poor behavioral outcomes, such as social functioning, resilience, and medication adherence [17, 20, 46]. The finding that enacted HIV stigma was negatively associated with environmental QoL is consistent with studies among other adult populations showing the negative impact of HIV-related stigma in other aspects of QoL, such as physical health- and mental health-related QoL [18, 47, 48]. Results were robust across a range of sensitivity analyses considering measurement of QoL.

Anticipated HIV stigma was not associated with environmental QoL in the adjusted analyses, which diverges from previous findings linking anticipated HIV stigma to poor psychological-environmental QoL among adolescents living with HIV [49]. The differences in these results may be explained by Durban having an established HIV prevention and care program for FSW, as well as participants coming to terms with their HIV status in a community with high HIV burden [3]. Thus, the participants engaged in the program may select for those who have overcome anticipated HIV stigma in seeking health services and mitigate its effects [50]. However, the association between enacted HIV stigma and environmental QoL indicates that there remains a need to address the underlying causes of HIV-related stigma in the broader health system and society in Durban.

The negative associations between environmental QoL and both anticipated and internalized sex work stigmas provide further insight into the health outcomes of FSW living with HIV. Lower environmental QoL is a particular concern for FSW; sex work-related factors, such as venue type or managers, mobility, and exposure to violence, are key elements in FSW’s physical environment and accessibility of resources [51, 52]. FSW experience stigmatization based on perceptions about their morality and bodily integrity [53], which affects how they engage in their work. Additionally, sex work stigma stems from multiple levels and manifests in forms such as policy, laws, and the public [54, 55], all of which can impact sex worker spaces. The lack of an association between enacted sex work stigma and environmental QoL may be attributed to local non-governmental organizations’ advocacy for FSW rights [56], as well as the study participants’ involvement with sex work venues. Social support from FSW peers may lessen the impact of enacted sex work stigma [47]. However, FSW are still vulnerable to biased and punitive encounters with law enforcement due to sex work criminalization [57]. Our study reported higher prevalence of sex work stigmas versus HIV stigmas, although the difference may be inflated due to the higher number of sex work stigma items in the baseline questionnaire. This higher prevalence was also observed in a Zimbabwe study [58]. Despite this common theme of sex work-related stigma among FSW [59, 60], few studies investigate this stigma attribute. A systematic study on evaluating stigma among sex workers found that only three percent of the sampled studies measured sex work-related stigmas [61]. This underscores the need to go beyond HIV-related stigmas and address sex work stigmas in the HIV epidemic.

Interventions should focus on removing drivers of stigma per Stangl and Earnshaw’s Health Stigma and Discrimination Framework, as well as target the multiple socio-ecological levels at which stigma marking occurs [25]. Removing drivers of stigma can include collaborating with law enforcement and policymakers to reduce punitive measures taken against sex work. This would reduce FSW’s vulnerability to violence and exploitation, as well as contribute to an overall safer environment in conducting sex work [62]. Additionally, increasing health care worker sensitization to sex worker needs and realities and continually improving programming tailored by and for FSW living with HIV would subsequently reduce barriers to meeting basic needs and accessing care. Given existing research on stigma and other HIV-related treatment and health outcomes, addressing stigma may also further support overall treatment efforts and decrease the risk of onward transmission [11–14].

The data suggest similar relationships between stigma and both environmental and overall QoL outcomes. The model using overall QoL showed that enacted sex work stigma, internalized sex work stigma and enacted HIV stigma were associated with a decrease in prevalence of high overall QoL. Another analysis using the same study population similarly linked internalized stigma with overall QoL using the EuroQoL five dimension, three-level questionnaire (EQ-5D-3L) [63]. This previous analysis also reported that increased internalized and experienced stigma were associated with a lower EuroQoL visual analogue scale (EQ-VAS). The impact on overall QoL further reinforces the need to intervene on both HIV and sex work-related stigmas experienced by FSW.

The associations between stigma and environmental QoL are corroborated by the sensitivity analysis examining the relationships between stigma and the other QoL domains. However, the low Cronbach’s alpha scores for the physical, psychological, level of independence, and SRPB domains (Additional file 2: Table S1) indicate the need for further scale development in the FSW population. Thus, the validity of these relationships and the QoL scale items should be explored in future research. On the other hand, the social relationships domain had a Cronbach’s alpha score of 0.72, and the analysis identified negative associations between stigma and this domain that were parallel to environmental QoL. Overall, the sensitivity analyses highlight analogous trends across different aspects of FSW’s QoL.

This study’s main strength is that the results contribute new information on the association between sex work-related stigmas and environmental QoL. This will inform interventions that reduce structural barriers to the well-being of FSW living with HIV beyond ART treatment and viral load suppression [27, 34]. Given the complexities of examining multiple sex work and HIV-related stigmas, more research is required to explore how the stigmas operate on QoL in relation to each other. There are also a variety of sex work environments depending on geographic and political settings [64], which can result in FSW experiencing differential barriers to HIV care and sources of support [65]. Hence, we recommend further investigation of stigma and QoL across various settings to identify common themes and points of intervention that could benefit a broader sex worker population.

There are several limitations that need to be considered. Primarily, this study was a cross-sectional analysis of stigma and QoL. Therefore, it is difficult to infer a temporal relationship. Second, both stigma and QoL are challenging to measure as they are subjective to individual experience. In particular, there is no single scale that is commonly used to measure sex work-related stigmas [61]. Thus, the inferences or the strengths of the associations might differ had other measurement tools been used. Additionally, these measures may have been subject to recall bias or social response bias, where individuals may misreport responses to appear more socially desirable [66]. Finally, although we anticipate that the results likely could generalize to the sex work community living with HIV in eThekwini and potentially other urban areas in South Africa with experienced programming efforts to serve FSW, the findings may not be generalizable to sex workers living in other metropolitan areas or who have not been provided with HIV prevention and care services.

## Conclusions

FSW require more attention in global efforts to mitigate the HIV epidemic. This study provides new evidence that FSW’s life outcomes are impacted by stigmas resulting from the unique positioning of their occupation and HIV infection status. Specifically, we demonstrate that stigma is linked with environmental QoL, a key aspect of the wellbeing of FSW living with HIV. More targeted efforts to reduce stigma among healthcare practitioners, the police, and broader society are needed, alongside efforts to promote physical safety and well-being of FSW. The drivers of sex work- and HIV-related stigmas can be lessened via educational programs, tackling policy barriers, and more intentional delivery of HIV treatment services among the FSW population [1, 65]. Structural changes addressing stigma are challenging, but they offer promise given stigma’s impact on numerous health-related outcomes [11–14]. By developing holistic interventions in partnership with FSW community leadership, a more comprehensive approach to addressing HIV treatment needs can emerge and ensure that quality of life is not forgotten in the process.

## Supplementary Information

**Additional file 1: Table A.** Stigma items and corresponding subscale. **Table B.** WHO Quality of Life-HIV Brief (WHOQOL-HIV BREF) Questionnaire Environmental Domain Items.

**Additional file 2: Table S1.** WHO Quality of Life–HIV Brief Questionnaire domain scores in FSW living with HIV (n = 1373) **Table S2.** One-Way ANOVA of WHO Quality of Life-HIV Brief Questionnaire domain mean scores. **Table S3.** Adjusted quality of life mean score difference in FSW living with HIV by stigma (n = 1373). **Table S4.** Adjusted prevalence ratios of higher quality of life in FSW living with HIV (n = 1373).

## Data Availability

The dataset used and analyzed during the current study are available from the corresponding author on reasonable request and approval.

## Abbreviations

FSW: Female sex workers
HIV: Human immunodeficiency virus
QoL: Quality of life
WHO: World Health Organization
ART: Antiretroviral therapy
SRPB: Spirituality/religion/personal beliefs
ZAR: South African rand
PR: Prevalence ratio
VIF: Variance inflation factor
EQ-5D-3L: EuroQol five dimension, three-level questionnaire
EQ-VAS: EuroQoL visual analogue scale
