# Supplementary material for: HIV- and sex work-related stigmas and quality of life of female sex workers living with HIV in South Africa: a cross-sectional study
Source: BMC Infect Dis. 2022 Dec 6;22:910. doi: 10.1186/s12879-022-07892-4 (PMC9724359; doi:10.1186/s12879-022-07892-4)
Supplement: Supplementary file 1 — Additional file 1: Table A. Stigma items and corresponding subscale. Table B. WHO Quality of Life-HIV Brief (WHOQOL-HIV BREF) Questionnaire Environmental Domain Items. [file 12879_2022_7892_MOESM1_ESM.docx]

**APPENDIX**

**Table A.** Stigma items and corresponding subscale

| **Stigma Question** | **Attribute** | **Subscale** |
| --- | --- | --- |
| Have you ever been afraid to seek healthcare because someone might learn that you are living with HIV? | HIV | Anticipated |
| Have you ever avoided seeking healthcare because you were afraid someone might learn that you are living with HIV? | HIV | Anticipated |
| Have you ever been afraid to be in public places because you are living with HIV? | HIV | Anticipated |
| Have you ever felt that you were blackmailed because you are living with HIV? | HIV | Anticipated |
| Have you ever been afraid to seek healthcare because someone might learn that you are a sex worker? | Sex Work | Anticipated |
| Have you ever avoided seeking healthcare because you were afraid someone might learn that you are a sex worker? | Sex Work | Anticipated |
| Have you ever been afraid to be in public places because you are FSW? | Sex Work | Anticipated |
| Have you ever avoided carrying condoms because you thought they might cause you problems from a uniformed officer? | Sex Work | Anticipated |
| Have you ever felt that you were blackmailed because you are FSW? | Sex Work | Anticipated |
| Have you ever been denied health services or have someone keep you from receiving health services because you are living with HIV? | HIV | Enacted |
| Have you ever felt that you were verbally harassed because you are living with HIV? | HIV | Enacted |
| Have you ever felt excluded from family activities because you are living with HIV? | HIV | Enacted |
| Have you ever felt that a family member made a negative remark or gossiped about you because you are living with HIV? | HIV | Enacted |
| Have you ever felt rejected by your friends because you are living with HIV? | HIV | Enacted |
| Have you ever felt that you were not treated well in a healthcare center because you are living with HIV? | HIV | Enacted |
| Have you ever felt that a healthcare worker made negative remarks or gossiped about you because you are living with HIV? | HIV | Enacted |
| Have you ever been denied health services or have someone keep you from receiving health services because you are a sex worker? | Sex Work | Enacted |
| Have you ever felt that you were verbally harassed because you’re FSW? | Sex Work | Enacted |
| Have you ever been arrested on charges related to prostitution? | Sex Work | Enacted |
| Have you ever felt excluded from family activities because you are a sex worker? | Sex Work | Enacted |
| Have you ever felt that a family member made a negative remark or gossiped about you because you are a sex worker? | Sex Work | Enacted |
| Have you ever felt rejected by your friends because you are a sex worker? | Sex Work | Enacted |
| Have you ever felt that you were not treated well in a healthcare center because you are a sex worker? | Sex Work | Enacted |
| Have you ever felt that a healthcare worker made negative remarks or gossiped about you because you are a sex worker? | Sex Work | Enacted |
| Have you ever felt that a uniformed officer refused to protect you because you are a sex worker? | Sex Work | Enacted |
| Have you ever felt a uniform officer harassed or intimidated you because you are a sex worker? | Sex Work | Enacted |
| Selling sex is a satisfactory and acceptable way of life for me. | Sex Work | Internalized |
| For the most part, I do not care if people know I sell sex. | Sex Work | Internalized |
| Selling sex does not make me a lesser person. | Sex Work | Internalized |

**Table B.** WHO Quality of Life – HIV Brief (WHOQOL-HIV BREF) Questionnaire Environmental Domain Items

| **Question** | **Response** |
| --- | --- |
| “The following questions ask about how much you have experienced certain things in the last TWO weeks.” | |
| How safe do you feel in your daily life? | 01 Not at all  02 A little  03 A moderate  04 Very Much  05 Extremely  88 Refusal |
| How healthy is your physical environment? | 01 Not at all  02 A little  03 A moderate  04 Very Much  05 An extreme amount  88 Refusal |
| “The following questions ask about how completely you experience or were able to do certain things in the last TWO weeks.” | |
| Have you enough money to meet your needs? | 01 Not at all  02 A little  03 Moderately  04 Mostly  05 Completely  88 Refusal |
| How available to you is the information that you need in your day-to-day life? | 01 Not at all  02 A little  03 Moderately  04 Mostly  05 Completely  88 Refusal |
| To what extent do you have the opportunity for leisure activities? | 01 Not at all  02 A little  03 Moderately  04 Mostly  05 Completely  88 Refusal |
| “The following questions ask you how good or satisfied you have felt about various aspects of your life over the last TWO weeks.” | |
| How satisfied are you with the conditions of your living place? | 01 Very dissatisfied  02 Dissatisfied  03 Neither dissatisfied nor satisfied  04 Satisfied  05 Very satisfied  88 Refusal |
| How satisfied are you with your access to health services? | 01 Very dissatisfied  02 Dissatisfied  03 Neither dissatisfied nor satisfied  04 Satisfied  05 Very satisfied  88 Refusal |
| How satisfied are you with your transport? | 01 Very dissatisfied  02 Dissatisfied  03 Neither dissatisfied nor satisfied  04 Satisfied  05 Very satisfied  88 Refusal |
