# Supplementary material for: HIV- and sex work-related stigmas and quality of life of female sex workers living with HIV in South Africa: a cross-sectional study
Source: BMC Infect Dis. 2022 Dec 6;22:910. doi: 10.1186/s12879-022-07892-4 (PMC9724359; doi:10.1186/s12879-022-07892-4)
Supplement: Supplementary file 2 — Additional file 2: Table S1. WHO Quality of Life–HIV Brief Questionnaire domain scores in FSW living with HIV (n = 1373) Table S2. One-Way ANOVA of WHO Quality of Life-HIV Brief Questionnaire domain mean scores. Table S3. Adjusted quality of life mean score difference in FSW living with HIV by stigma (n = 1373). Table S4. Adjusted prevalence ratios of higher quality of life in FSW living with HIV (n = 1373). [file 12879_2022_7892_MOESM2_ESM.docx]

**ADDITIONAL TABLES**

**Table S1.** WHO Quality of Life–HIV Brief Questionnaire domain scores in FSW living with HIV (n=1373)

| **Domain** | **Mean Score (SD)** | **Cronbach’s Alpha** |
| --- | --- | --- |
| Physical | 13.54 (3.44) | 0.42 |
| Psychological | 13.76 (2.69) | 0.58 |
| Level of Independence | 13.79 (2.87) | 0.45 |
| Social Relationships | 12.76 (3.40) | 0.72 |
| Environment | 10.54 (2.73) | 0.70 |
| Spirituality/religion/personal beliefs (SRPB) | 13.61 (3.41) | 0.49 |

**Table S2.** One-Way ANOVA of WHO Quality of Life – HIV Brief Questionnaire domain mean scores

| **Domain Mean Scores for Comparison** | **Domain Mean Score Difference (95% CI)** | **p-value** |
| --- | --- | --- |
| Psychological – Physical | 0.22 (-0.12, 0.56) | 0.423 |
| Level of Independence – Physical | 0.25 (-0.09, 0.59) | 0.287 |
| Social Relationships – Physical | -0.78 (-1.12, -0.44) | <0.001 |
| Environment – Physical | -3.00 (-3.34, -2.66) | <0.001 |
| SRPB – Physical | 0.07 (-0.26, 0.41) | 0.989 |
| Level of Independence – Psychological | 0.03 (-0.31, 0.37) | 0.999 |
| Social Relationships – Psychological | -1.00 (-1.34, -0.66) | <0.001 |
| Environment – Psychological | -3.22 (-3.56, -2.88) | <0.001 |
| SRPB – Psychological | -0.15 (-0.48, 0.19) | 0.816 |
| Social Relationships - Level of Independence | -1.03 (-1.37, -0.69) | <0.001 |
| Environment – Level of Independence | -3.25 (-3.59, -2.91) | <0.001 |
| SRPB – Level of Independence | -0.17 (-0.51, 0.16) | 0.680 |
| Environment – Social Relationships | -2.22 (-2.56, -1.88) | <0.001 |
| SRPB – Social Relationships | 0.85 (0.52, 1.19) | <0.001 |
| SRPB – Environment | 3.07 (2.74, 3.41) | <0.001 |

**Table S3.** Adjusted quality of life mean score difference in FSW living with HIV by stigma (n=1373)

| **Attribute** | **Stigma Subscale** | **Environmental QoL** | | **Overall QoL** | |
| --- | --- | --- | --- | --- | --- |
|  |  | **Mean Difference^*^ (95% CI)** | **p-value** | **Mean Difference^*^ (95% CI)** | **p-value** |
| Sex Work | **Any Anticipated** (ref: no anticipated) | -0.90 (-1.24, -0.56) | <0.001^**^ | -0.11 (-0.26, 0.04) | 0.143 |
|  | **Any Enacted**  (ref: no enacted) | -0.14 (-0.47, 0.20) | 0.418 | -0.18 (-0.33, -0.03) | 0.020^**^ |
|  | **Internalized**  None/minimal  (ref: 3-6) | REF |  | REF |  |
|  | Mild (7-9) | -0.24 (-0.61, 0.13) | 0.201 | -0.01 (-0.17, 0.16) | 0.932 |
|  | Moderate (10-12) | -0.51 (-0.85, -0.16) | 0.005^**^ | -0.26 (-0.42, -0.11) | <0.001^**^ |
|  | Severe (13-15) | -1.24 (-1.71, -0.78) | <0.001^**^ | -0.37 (-0.58, -0.17) | <0.001^**^ |
| HIV | **Any Anticipated** (ref: no anticipated) | -0.04 (-0.60, 0.52) | 0.895 | -0.16 (-0.41, 0.09) | 0.220 |
|  | **Any Enacted**  (ref: no enacted) | -0.59 (-1.00, -0.19) | 0.004^**^ | -0.26 (-0.44, -0.08) | 0.005^**^ |
| Mean score differences were estimated using linear regression as a sensitivity analysis.  ^*^Adjusted for age, level of education, monthly income, length of HIV diagnosis  ^**^p-value <0.05 | | | | | |

**Table S4.** Adjusted prevalence ratios of higher quality of life in FSW living with HIV (N=1373)

| **Attribute** | **Stigma Subscale** | **Higher Physical QoL** | | **Higher Psychological QoL** | | **Higher Level of Independence QoL** | | |
| --- | --- | --- | --- | --- | --- | --- | --- | --- |
|  |  | **aPR^*^ (95% CI)** | **p-value** | **aPR^*^ (95% CI)** | **p-value** | **aPR^*^ (95% CI)** | **p-value** | |
| Sex Work | **Any Anticipated** (ref: no anticipated) | 0.80 (0.73, 0.87) | <0.001^**^ | 0.92 (0.86, 0.98) | 0.011^**^ | 0.87 (0.82, 0.93) | <0.001^**^ | |
|  | **Any Enacted**  (ref: no enacted) | 1.07 (1.00, 1.62) | 0.067 | 0.98 (0.92, 1.04) | 0.462 | 1.02 (0.97, 1.08) | 0.435 | |
|  | **Internalized**  None/minimal  (ref: 3-6) | REF |  | REF |  | REF |  | |
|  | Mild (7-9) | 0.90 (0.83, 0.99) | 0.033^**^ | 0.97 (0.91, 1.03) | 0.351 | 0.94 (0.88, 1.00) | 0.060 | |
|  | Moderate (10-12) | 0.94 (0.87, 1.02) | 0.132 | 0.85 (0.79, 0.91) | <0.001^**^ | 0.95 (0.90, 1.01) | 0.111 | |
|  | Severe (13-15) | 0.88 (0.77, 0.99) | 0.038^**^ | 0.78 (0.69, 0.88) | <0.001^**^ | 0.68 (0.59, 0.78) | <0.001^**^ | |
| HIV | **Any Anticipated** (ref: no anticipated) | 0.91 (0.78, 1.07) | 0.271 | 1.05 (0.93, 1.18) | 0.442 | 0.82 (0.72, 0.95) | 0.008^**^ | |
|  | **Any Enacted**  (ref: no enacted) | 1.02 (0.92, 1.13) | 0.715 | 0.92 (0.84, 1.00) | 0.056 | 1.00 (0.92, 1.08) | 0.945 | |
| Adjusted prevalence ratios (aPR) were estimated using modified robust Poisson regression.  *Adjusted for age, level of education, monthly income, length of HIV diagnosis  **p-value <0.05 | | | | | |  | |  |

| **Attribute** | **Stigma Subscale** | **Higher Social Relationships QoL** | | **Higher Spirituality/Religion/Personal Beliefs (SRPB) QoL** | |
| --- | --- | --- | --- | --- | --- |
|  |  | **aPR^*^ (95% CI)** | **p-value** | **aPR^*^ (95% CI)** | **p-value** |
| Sex Work | **Any Anticipated**  (ref: no anticipated) | 0.86 (0.79, 0.94) | <0.001^**^ | 0.82 (0.75, 0.88) | <0.001^**^ |
|  | **Any Enacted**  (ref: no enacted) | 0.97 (0.90, 1.04) | 0.412 | 1.07 (1.00, 1.15) | 0.050 |
|  | **Internalized**  None/minimal  (ref: 3-6) | REF |  | REF |  |
|  | Mild (7-9) | 0.92 (0.84, 1.00) | 0.058 | 0.98 (0.91, 1.06) | 0.692 |
|  | Moderate (10-12) | 0.84 (0.77, 0.92) | <0.001^**^ | 0.92 (0.85, 0.99) | 0.046^**^ |
|  | Severe (13-15) | 0.67 (0.57, 0.78) | <0.001^**^ | 0.79 (0.70, 0.91) | <0.001^**^ |
| HIV | **Any Anticipated**  (ref: no anticipated) | 1.13 (0.94, 1.33) | 0.132 | 0.95 (0.82, 1.11) | 0.525 |
|  | **Any Enacted**  (ref: no enacted) | 0.73 (0.64, 0.83) | <0.001^**^ | 1.00 (0.91, 1.10) | 0.985 |
| Adjusted prevalence ratios (aPR) were estimated using modified robust Poisson regression.  *Adjusted for age, level of education, monthly income, length of HIV diagnosis  **p-value <0.05 | | | | | |
